# Evaluation of a temperature-responsive magnetotocosome as a magnetic targeting drug delivery system for sorafenib tosylate anticancer drug

**DOI:** 10.1016/j.heliyon.2023.e21794

**Published:** 2023-11-04

**Authors:** Fariba Razmimanesh, Gholamhossein Sodeifian

**Affiliations:** a Department of Chemical Engineering, Faculty of Engineering, University of Kashan, 87317-53153, Kashan, Iran; b Laboratory of Biotechnology and Nanotechnology, University of Kashan, 87317-53153, Kashan, Iran; c Biotechnology Centre, Faculty of Engineering, University of Kashan, 87317-53153, Kashan, Iran

**Keywords:** Magnetotocosome, Biomaterial, Blend of PNIPAAm and chitosan, LCST, RAFT polymerization method, Molecular weight effect

## Abstract

In this investigation, a polymeric fusion of chitosan (CS) and thermosensitive poly (N-isopropyl acrylamide) - PNIPAAm - encapsulated a magnetotocosome, biocompatible nanocarrier. This encapsulation strategy demonstrated improved drug entrapment efficiency, achieving up to 98.8 %. Additionally, it exhibited extended stability, optimal particle dimensions, and the potential for industrial scaling, thus facilitating controlled drug delivery of sorafenib tosylate to cancerous tissue. Reversible Addition-Fragmentation Chain Transfer (RAFT) techniques were employed to synthesize PNIPAAm. The effects of polymer molecular weight and polydispersity index on the lower critical solution temperature (LCST) were evaluated. The resulting polymeric amalgamation, involving the thermosensitive PNIPAAm synthesized using RAFT techniques and CS that coated the magnetotocosome (CS-Raft PNIPAAm-magnetotocosome) with an LCST approximately at 45 °C, holds the potential to enhance drug bioavailability and enable applications in hyperthermia treatment, controlled release, and targeted drug delivery.

## Highlights

• Temperature-responsive magnetotocosome was developed as the efficient drug delivery system for SFB.
• The effects of tocosome composition and drug concentration on particle size and drug loading were investigated.
• The effect of PC on the properties of SPIO NPs was studied.
• PNIPAAm was synthesized by two methods, including free radical and RAFT.
• Effects of MW and chain length of the polymers on LCST were examined.

## Graphical abstract

**Figure:**
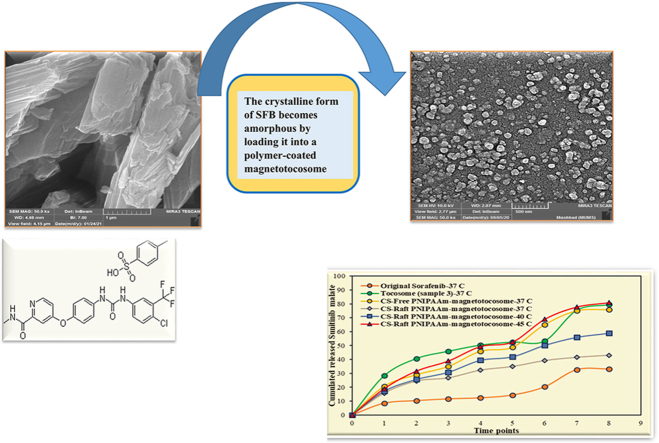

## 1 Introduction

**Fig. 1:**
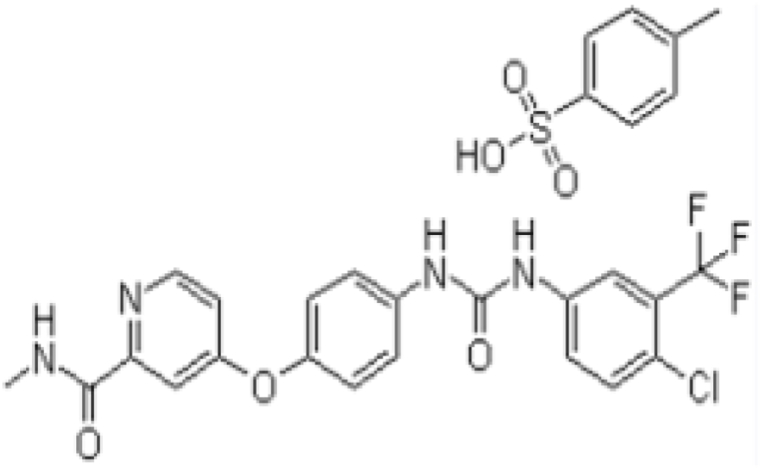
SFB chemical structure [4].

Renal cell carcinoma, the third most prevalent urologic malignancy, accounts for approximately 270,000 new cases annually, contributing to over 116,000 fatalities [1]. On a global scale, hepatocellular carcinoma is the fifth most prevalent malignant tumor and ranks as the third-highest cause of mortality [2,3]. The approval of sorafenib tosylate (SFB) marked a significant milestone, designating it as the foremost anticancer drug for managing hepatocellular carcinoma and renal cell carcinoma. The chemical structure of SFB is depicted in Fig. 1.

However, by the biopharmaceutical classification scheme, SFB is categorized as a class II compound due to its notably poor solubility across a spectrum of pH levels spanning pH 1.2 to pH 7.4. This characteristic results in a sluggish dissolution rate within the gastrointestinal tract, however its high permeability through the gastrointestinal lumen. The drug exhibits a low oral bioavailability of 8.43 %, necessitating elevated dosages for effective treatment. This elevated dosing regimen can give rise to systemic toxicity and complications, encompassing skin reactions, hypertension, alterations in thyroid hormone levels, bleeding, fatigue, and gastrointestinal disturbances. Consequently, the therapeutic efficacy of SFB becomes circumscribed [[1], [2], [3],[5], [6], [7], [8]]. Especially pertinent to compounds with a restricted therapeutic index or cytotoxic properties, the utilization of nanotechnology-based inventive drug delivery systems has gained traction. These systems are tailored to exhibit heightened biocompatibility and biodegradability, facilitating the secure and effective transport of drug molecules to target tissues and concurrently mitigating undesired side effects [1,3,5,9]. Limited endeavors have been undertaken to enhance the solubility and bioavailability of SFB. These pursuits include techniques such as encapsulation within poly-DL-lactide-co-glycolide (PLGA), PLGA/dextran copolymer, solid lipid nanoparticles (NPs), or liposomes, as well as complex formation with albumin, cyclodextrin, and dual polymeric-lipid NPs [2,3,[5], [6], [7], [8]]. Within the scope of pH-sensitive drug delivery systems, a noteworthy instance is the deployment of carboxymethyl chitosan coated cationic liposomal platform, effectively loaded with both SFB and siRNA. Notably, this liposomal arrangement presents a viable alternative for tumor therapy, given its negligible in vitro and in vivo toxicity [10]. Additionally, a study by Zhang et al. explored the development of magnetic folate-functionalized polymeric micelles configured for targeted delivery, contingent upon the presence of folate molecules, of SFB. The inhibitory potential of this drug delivery system was evaluated against human hepatic carcinoma cells in vitro. The evaluation of the efficacy of this targeted therapeutic approach was facilitated through magnetic resonance imaging [2]. By employing a process of hot homogenization, superparamagnetic magnetite (Fe_3_O_4_, SPIO) NPs, and SFB were encapsulated within cetyl palmitate solid lipid NPs. Comprehensive characterization of the resulting nanocarrier encompassed analyses of size distribution, morphology, zeta potential, drug loading, SPIO loading efficiency, and magnetic properties. Upon scrutiny of the acquired data, it can be deduced that the engineered stable NPs can proficiently and selectively transport the drug to the tumor site utilizing an external magnetic field, thereby impeding the proliferation of cancer cells [5]. The amalgamation of inorganic and organic constituents at the nanoscale has garnered significant attention due to its potential applications within medical diagnostics and therapeutics [[11], [12], [13]]. Notably, SPIO, characterized by its diminutive size (below 20 nm), emerges as a promising magnetic material with prospects spanning biomedical and biotechnological realms. This material finds applications in hyperthermia, physical therapy, magnetic resonance imaging, photoablation therapy, and targeted drug delivery [12,[14], [15], [16], [17]]. Superparamagnetism's distinctive physical attributes are contingent upon factors such as shape, size, and chemical composition. Additionally, owing to their diminished coercive field and remnant magnetization, these particles display reduced tendencies for agglomeration after applying a magnetic field. This property augments their mobility within the bloodstream and enhances their expulsion from the body. Long-chain capping molecules encompassing polymeric materials, surfactants, and thiol functional groups can be introduced to counteract the propensity for metal ion precipitation [18]. Introducing phospholipid molecules, such as soy phosphatidylcholine (soy-PC), which serve as liposome construction precursors can exert influence over magnetite nucleation and growth patterns [19].

**Fig. 2:**
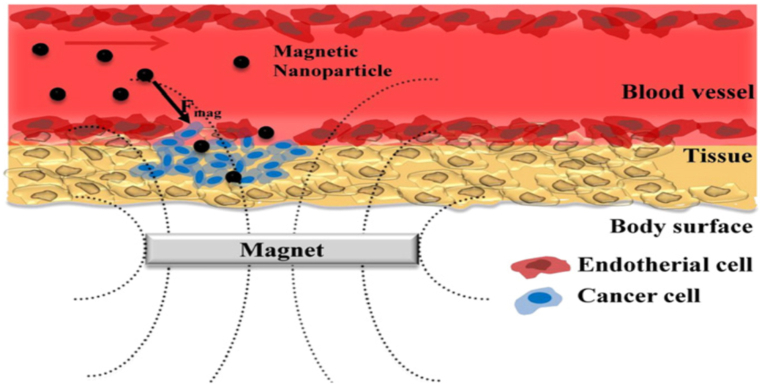
Schematic representation of a magnetic NPs-based drug delivery system [21].

Intelligent magnetic NPs adopt a core/shell configuration, wherein iron cores are enveloped by responsive moieties in the form of shells, often composed of polymers or liposomes [16]. These constructs facilitate the conveyance of drug molecules to cancer cells by applying an external magnetic field (Fig. 2). Moreover, compelling evidence supports the notion that magnetic hyperthermia, a technique involving the localized elevation of temperature within cancerous tissues, constitutes an effective strategy to induce apoptotic pathways in cancer cells [2,9,11,13,17,20].

Liposomes represent an esteemed and innovative drug delivery system for their elevated biocompatibility, minimal toxicity, and capacity to efficiently transport drugs to designated sites of action [[22], [23], [24], [25], [26], [27]]. The incorporation of PEG-derived lipids, such as PEG-DSPE, can strengthen the stability of colloidal liposomes. These sterically stabilized liposomes (SSL, Stealth) exhibit prolonged circulation half-lives, reduced uptake by the mononuclear phagocyte system, and heightened accumulation within tumors [25,26,28].

In encapsulation and controlled release of therapeutic agents, α-tocopheryl phosphate (TP) emerges due to its recognized anti-atherogenic, anti-inflammatory, cardioprotective, and anti-tumor invasion attributes [29]. The utilization of TP molecules in the construction of liposomes, termed tocosomes, has been evaluated by Mozafari et al. [29], yielding notably positive stability outcomes. This innovative methodology was harnessed to formulate tocosomes tailored for administering 5-fluorouracil, showcasing attributes like controlled size distribution, commendable encapsulation efficiency, and prolonged stability [23,27]. Importantly, Mozafari's approach, characterized by scalability and robustness, circumvents the use of toxic solvents or detergents throughout the synthesis process [29,30]. In this context, temperature-sensitive liposomes are a cutting-edge technique, offering substantial stability under physiological conditions (T = 37 °C) [10,31]. The thermoresponsive polymer poly (N-isopropyl acrylamide) (PNIPAAm) has garnered extensive exploration over the years, evident from the extensive body of literature devoted to this polymer [11,[32], [33], [34], [35], [36], [37], [38], [39]]. Predicated upon the precise macromolecular microstructure, PNIPAAm exhibits a lower critical solution temperature (LCST), typically ranging from 30 to 35 °C, contingent upon its concentration and molecular weight (MW).

The reversible and swift thermosensitivity of PNIPAAm, biocompatibility, and close-to-body LCST positions it as a highly desirable material for a spectrum of biomedical applications [[32], [33], [34],[40], [41], [42], [43], [44], [45], [46], [47]]. Furthermore, crafting magnetic NPs through the integration of PNIPAAm - a process involving encapsulating iron oxide magnetic cores within PNIPAAm shells - represents a pragmatic avenue for generating thermo-responsive magnetic nanocomposites [15,20].

Systems encompassing the amalgamation of liposomes within the chitosan (CS) biopolymer framework have demonstrated promising outcomes in targeted drug delivery and tissue engineering. The macromolecular architecture of CS features surface functional groups, notably amino and hydroxyl groups, which foster an elevated affinity for drug adsorption [1,12,13,20,48,32,[49], [50], [51], [52], [53], [54]]. On a contrasting note, the limited biodegradability of PNIPAAm stands as a primary constraint. Nonetheless, the mitigation of this limitation could be achieved through the creation of copolymers or the incorporation of N-isopropyl acrylamide (NIPAM) into short chains via alternative biodegradable polymers such as CS, collagen, hyaluronic acid, or other natural polymers, thereby presenting a prospective solution [34].

A balance between polymer-polymer and polymer-solvent interactions governs the LCST of PNIPAAm. Thus, manipulating PNIPAAm's transition temperature is attainable through MW, chain length, and concentration adjustments. Furthermore, its transition temperature can be influenced by copolymerization with other polymers or through amalgamation with surfactants and co-solvents [20,32,33,49]. Reversible addition-fragmentation chain transfer (RAFT) polymerization is an effective technique for crafting distinct, well-defined polymers with minimal polydispersity and MW variability, which is particularly valuable for biomedical applications [32,55,56].

In this study, a novel biocompatible nanocarrier was developed, which integrates magnetotocosome (tocosome with superparamagnetic iron oxide core) with a polymeric blend comprising thermosensitive PNIPAAm and CS, resulting in a temperature-responsive tocosome. This innovative nanocarrier is designed for the magnetic targeting delivery of SFB to cancerous tissues, thereby contributing to advancements in drug delivery strategies.

This system was meticulously crafted to enhance drug bioavailability, minimize the required drug dosage, mitigate toxicity toward healthy tissues or organs, and improve overall patient satisfaction. Integrating magnetotocosomes with polymers represents an innovative approach to optimizing drug delivery outcomes. A detailed investigation into SFB's characterization and controlled release in vitro has been executed through an array of analytical techniques, encompassing dynamic light scattering (DLS), ^1^H Nuclear magnetic resonance (^1^H NMR), Fourier-transform infrared spectroscopy (FTIR), differential scanning calorimetry (DSC), field-emission scanning electron microscopy (FE-SEM), gel permeation chromatography (GPC), vibrating sample magnetometer (VSM), X-ray diffraction (XRD), and ultraviolet–visible (UV–Vis) spectroscopy. The method established by Mozafari, known for its scalability and robustness, obviates the need for organic solvents during tocosome preparation. The pursuit of magnetotocosome development involved a comprehensive exploration of the impacts of cholesterol (CHL) molar ratio and drug concentration on critical parameters such as particle size, zeta potential, encapsulation effectiveness (EE%), and drug loading within the tocosome framework. Subsequently, these findings were synergistically integrated within a polymeric matrix. Additionally, the influence of phosphatidylcholine (PC) on SPIO nanoparticle synthesis and characteristics was subject to scrutiny. The PNIPAAm homopolymer was synthesized through free radical and RAFT polymerization methodologies. Regarding the LCST, an assessment was conducted on the influences of polymer MW, chain length, and polydispersity index (PDI). The developed drug-delivery system retains its capacity even when temperatures exceed physiological norms.

## 2 Experimental sections

### 2.1 Materials

CHL, soy-PC, TP, DSPE-PEG (2000) amine, NIPAM with a purity of 97 %, medium molecular weight CS (deacetylation degree of 85 %), benzyl benzodithioate (96 %) employed as the RAFT agent for controlled radical polymerization, azobisisobutyronitrile (AIBN, 98 %), sodium bisulfite (SBS, 99 %), ammonium persulfate (APS, 98 %), iron (III) chloride hexahydrate (FeCl_3_·6H_2_O, 98 %), iron (II) chloride tetrahydrate (FeCl_2_·4H_2_O, 99 %), all of reagent grade, and ammonium hydroxide (28 % w/w NH_3_) were sourced from Sigma Aldrich USA. Analytical-grade chemicals such as methanol, 1,4-dioxane, hexane, glacial acetic acid (99 %), and pentane were obtained from the Merck Company (Darmstadt, Germany). The SFB used in the study was acquired from a Persian pharmaceutical firm. NIPAM and AIBN were subjected to two crystallization processes in hexane and methanol. All other substances, including solvents, were employed without further purification.

### 2.2 Method

#### 2.2.1 Tocosome preparation

Following the approach detailed in Table 1, the tocosomal drug delivery systems, as outlined in Mozafari's method [57], were fashioned by combining soy-PC, TP, DSPE-PEG (2000) amine, and CHL. The process was initiated with the dissolution of CHL in a solution of phosphate-buffered saline (PBS, pH 7.4) along with glycerol (final concentration of 3 % v/v). Stirring at approximately 1200 rpm was maintained in a nitrogen atmosphere at elevated temperatures (approximately 125 °C) for 45 min. The components for tocosome formulation including soy-PC, TP, DSPE-PEG (2000) amine with the SFB drug were added to CHL and glycerol mixture. The temperature was then lowered to 65 °C, above the lipid's phase transition temperature. The system was stirred at around 1000 rpm and heated to 65 °C for 60 min within a nitrogen environment. After this stage, the tocosomal samples underwent filtration using a 0.42 μm syringe filter.

**Table 1:** Particle size, zeta potential, encapsulation efficiency (EE%), and loading (DL%) of SFB drug into tocosome and polymeric blends (CS and PNIPAAm)-coated magnetotocosome, variation in particle size of samples after three months (d_3_-d_0_).

Sample	Tocosome Composition	Molar ratio	The mass ratio (drug/lipid)	Mean particle size (nm) ± SD^a^	DL%±SD	EE%±SD	Zeta potential (mV) ± SD	d_3_-d_0_±SD
1	TP:PC:DSPE-PEG(2000):CHL	3:7:0.28:4	1/20	127.2 ± 2.8	2.59 ± 0.10	51.61 ± 2.1	−5±0.1	2.5 ± 0.1
2	TP:PC:DSPE-PEG(2000):CHL	3:7:0.28:0.8	1/20	64.9 ± 2.3	3.52 ± 0.11	70.32 ± 2.3	−11.9 ± 0.3	3.7 ± 0.1
3	TP:PC:DSPE-PEG(2000):CHL	3:7:0.28:0.8	1/8	75.9 ± 2.6	9.46 ± 0.15	75.69 ± 1.3	−19.1 ± 0.5	4.5 ± 0.2
4	TP:PC:DSPE-PEG(2000):CHL	3:7:0.28:4	1/8	147.8 ± 4.7	9.75 ± 0.12	77.88 ± 1.1	−14.7 ± 0.2	2.9 ± 0.1
CS-Free PNIPAAm-magnetotocosome	–	–	–	347.8 ± 7.1	1.21 ± 0.02	97.2 ± 2.5	−13.3 ± 0.3	9.1 ± 0.3
CS-Raft PNIPAAm-magnetotocosome	–	–	–	314.5 ± 6.8	1.24 ± 0.03	98.8 ± 2.9	−15 ± 0.4	15.3 ± 0.6

The ensuing tocosomal samples were subjected to annealing and stabilization at room temperature, sustained in a nitrogen atmosphere for 60 min. To eliminate unencapsulated drugs, centrifugation was executed using a UNIVERSAL PIT 320 centrifuge at 12,000 rpm and 4 °C for 60 min. This step followed an 8-h incubation of the samples at 4 °C (achieved via two 30-min intervals). The final step involved using a bath sonicator (CODYSON CD-4820) to disintegrate the multilamellar vesicle suspension (in the remaining solution or pellet) into smaller vesicles, thereby reducing the number of bilayers. Each sample was subjected to three distinct preparation cycles.

#### 2.2.2 Magnetic synthesis

Coprecipitation is the optimal method for biomedical applications that necessitate finely controlled particle size distribution due to its ability to yield heightened crystallinity and saturation magnetization within a narrow range.

a Super paramagnetic Iron Oxide (SPIO)

In 40 mL of deionized water, 0.99 g (equivalent to 5 mmol) of FeCl_2_·4H_2_O and 2.7 g (equivalent to 10 mmol) of FeCl_3_·6H_2_O were thoroughly mixed. Subsequently, the temperature of the solution was raised to 85 °C. Upon reaching this temperature, the solution was exposed to ultrasonic waves, and within this nitrogen (N_2_) atmosphere, 18 mL of NH_4_OH (25 % solution in water) was gradually introduced drop by drop.

The reaction was sustained at 85 °C for 2 h. Following this, the mixture was allowed to cool down to room temperature. The resultant black sediments were then gathered utilizing magnets, and subsequently, they underwent filtration with ethanol and deionized water; this process was repeated four times to ensure the removal of any residual reactants. The sediments were then vacuum drying within a desiccator positioned over silica gel.

b PC-coated Superparamagnetic Iron Oxide (PC-SPIO)

The synthesis of PC-SPIO NPs was accomplished through coprecipitation. Initially, an aqueous solution containing 0.1 M FeCl_2_ and FeCl_3_ salts was prepared, maintaining a molar ratio of 2:1. For Methanol, three distinct PC concentrations were introduced, resulting in molar ratios of 0.5 %, 1.0 %, and 1.5 % of PC to iron ions (Fe^2+^ and Fe^3+^). This introduction of Methanol is aimed at enhancing the solubility of PC in water, thereby fostering direct interaction between PC and the magnetic particles. This strategic addition aids in reducing premature agglomeration during the crystallization process**.**

After combining the PC solution and the metal ion solution under agitation, the metal ions were precipitated by the incremental addition of NH_3_ (25 % solution in water) with vigorous stirring (at 1200 rpm). The reaction solution was heated at 85 °C for 2 h while continuously stirring. The process included Methanol evaporation to optimize the contact area between the newly formed particles and the surfactants.

Permanent magnets were employed to separate PC-SPIO NPs from the aqueous supernatant. To eliminate chloride ions, the resulting precipitates underwent a sequence of washes: five washes with cold water, followed by two washes with acetone, and ultimately, one wash with Methanol. A portion of the obtained PC-stabilized SPIO precipitates was air-dried to facilitate subsequent analyses.

#### 2.2.3 Magnetotocosome synthesis

The magnetotocosome was synthesized employing a procedure akin to the one delineated in the preceding section (tocosome preparation). However, in this iteration, dispersed SPIO NPs within PBS were incorporated into the mixture of CHL and glycerol, along with other tocosome constituents and the SFB drug. The integration of the SPIO NPs into the process occurred through sonication.

The selection of the optimal tocosome composition was paramount for this endeavor. Post magnetotocosome preparation was stored at 65 °C for 60 min within a nitrogen atmosphere. Subsequently, the solution underwent filtration using a 0.42 μm syringe filter. Notably, during this process, the unloaded drug molecules were not isolated from the solution, as they were intended to be subsequently loaded into the polymeric blend in the subsequent phase.

The ultimate step involved employing a bath sonicator to create smaller vesicles with diminished bilayer counts. This was achieved by disrupting a multilamellar vesicle suspension, ultimately facilitating the formation of smaller vesicles with fewer bilayers.

#### 2.2.4 PNIPAAm synthesis

The synthesis of the PNIPAAm polymer was accomplished through the utilization of both free radical and RAFT polymerization techniques. For free radical polymerization, the process was conducted at a temperature notably higher than PNIPAAm's LCST.

At an elevated temperature of 65 °C, NIPAM was subjected to polymerization through the free radical mechanism employing a sulfate initiator [33]. The procedure commenced by dissolving NIPAM (0.040 M) within 66 mL of water, which was subsequently degassed for 30 min under N_2_ environment. The polymerization reaction spanned 7 h at the same 65 °C temperature, employing vigorous stirring at a speed of 600 rpm. To initiate the polymerization, 3.5 mL of aqueous APS solution (0.01 M) was added, and the process was catalyzed by the addition of 3.5 mL of aqueous SBS solution (0.005 M).

The ensuing polymer was precipitated using a mixture of water and acetone in a 1:1 ratio. This mixture was used for precipitation after the solution had cooled to room temperature, approximately 25 °C. It's worth noting that, similar to polyacrylamide, PNIPAAm is insoluble in acetone-water mixtures but readily dissolves in pure acetone. This methodology yielded 0.14 g of polymer, accompanied by a NIPAM conversion rate of 46%. The conversion was calculated by difference between initial mass of monomer and prepared polymer mass (when the solvent was completely evaporated). But since the monomer peak was observed in the produced polymer, the percentage of monomer in the polymer was measured using the NMR results and the conversion percentage was taken into account.

The RAFT method employed the chain transfer agent (CTA) known as benzyl benzodithioate in combination with AIBN as the initiator. This strategy facilitated the creation of a low PDI and MW PNIPAAm polymer. The molar ratio of [NIPAM]/[CTA]/[AIBN] was maintained at 40/1/0.1 throughout this procedure.

The procedure was as follows: NIPAM (0.3 g), CTA (18.3 mg), AIBN (2.4 mg), and 1,4-dioxane (10 mL) were meticulously combined. Before reaching a temperature of 65 °C for 7 h, the solution underwent a deoxygenation process through N_2_ bubbling, executed for 30 min. The solution subsequently underwent vacuum evaporation.

The resulting polymer was recovered by precipitation in diethyl ether, followed by dissolution in tetrahydrofuran, reprecipitation in pentane, and thorough washing with pentane. The products were then subjected to vacuum drying, yielding a yellow powder. This process yielded 0.27 g (equivalent to 90%) of polymer. It was calculated by difference between initial mass of monomer and prepared polymer mass (when the solvent was completely evaporated).

The average molecular weights of the polymers were determined through GPC.

#### 2.2.5 Polymeric blend preparation

Polymeric blends were synthesized by dissolving PNIPAAm within an aqueous solution of CS (10 wt%) containing acetic acid. The solution underwent continuous agitation for 1 h to ensure thorough and uniform mixing of CS and PNIPAAm. Following this mixing step, the solution was transferred to a watch glass.

Following findings from a previous study [33], the blending ratio of the two components was adjusted to 30 wt% of CS about PNIPAAm. To create a film containing the CS/PNIPAAm composite, the solution was subjected to a temperature of 40 °C within a vacuum environment for 24 h. This step followed the evaporation of the solution at ambient temperature during the preceding night.

#### 2.2.6 Coated-magnetotocosome with polymeric blend

The polymeric blend was dissolved in PBS with a pH of 7.4 and stirred magnetically overnight at room temperature. The solutions underwent filtration through a 5 μm syringe filter to minimize the potential for dust and contaminated particles. Incorporating magnetotocosomes into the polymer solutions was conducted dropwise at a ratio of 1:5 while stirring for 5 min. Subsequently, the polymer-coated magnetotocosomes were subjected to overnight refrigeration (at 4 °C) to achieve stabilization.

For the generation of non-flocculating polymer-coated liposomes, a polymer concentration of 0.1 % (w/w) was employed [31]. The unencapsulated SFB was separated through centrifugation at 12,000 rpm. The resulting solutions underwent treatment in an ultrasonic bath and were stored at 4 °C for further analysis. Each specimen was subjected to this process three times to ensure consistency and reproducibility.

### 2.3 Characterization techniques

This study employed various characterization techniques, including UV–Vis spectroscopy, FTIR, XRD, ^1^H NMR, DLS, GPC, DSC, VSM, and FE-SEM, to thoroughly assess the manufactured nanocarriers. The following provides an overview of how these techniques were employed to evaluate aspects such as NP production, size distribution, stability, magnetization, and shape.

At a temperature of 25 °C, GPC analysis was performed using a refractive index (RI) detector and an ODS hypersil column (length 100, diameter 2.1, size 5). This allowed for determining the MW and polydispersity of PNIPAAm polymers. A mobile phase consisting of high-performance liquid chromatography (HPLC) grade tetrahydrofuran (THF) with 0.25 % (w/v) tetrabutylammonium bromide (TBABr) was employed for the analysis of PNIPAAm.

FTIR analysis was conducted on vacuum-dried samples to investigate interactions between magnetotocosome and polymeric blends. IR spectra were collected using an FTIR spectrophotometer (Magna 550, Nicolet Instruments Corporation, USA) employing the KBr pellet method. Wavelengths ranging from 400 cm^−1^–4000 cm^−1^ were utilized for data collection.

To determine the structures and chemical compositions of homopolymers, ^1^H NMR analysis was employed. A Bruker AVANCE DPX 300 NMR spectrometer operating at 300.13 MHz at 25 °C, with dimethyl sulfoxide as the solvent, was utilized. Chemical composition variations were expressed in parts per million (ppm).

The DLS technique was employed to characterize the samples' mean diameter and particle size distribution. This analysis used a Zetasizer Nano-ZS instrument (Malvern Instruments, UK). Tocosomes were dispersed in PBS, and their sizes were determined within a stable colloid environment at 25 °C. The viscosity of the medium was 1.02 cP, and the refractive index was 1.335.

These techniques collectively provided a comprehensive understanding of the nanocarriers' properties, enabling a thorough assessment of their performance and potential applications.Additionally, the surface charge of the NPs (zeta potential) was assessed to investigate the surface characteristics of tocosomes and the adsorption of polymers. The zeta potential was determined by calculating electrophoretic mobility (UE) using the Henry equation, considering parameters such as dielectric constant and water viscosity.

Calorimetric analyses were conducted using a DSC to explore the nanocarriers' properties further. The LCST was determined based on either the temperature corresponding to the endothermic peak on the DSC diagram or the point at which transmittance reaches 50 % on the transmittance-temperature curve [34,58].

For the analysis of specimen magnetization, the VSM technique was employed at room temperature with a field strength of 15,000 Oe (Meghnatis Daghigh Kavir Co., Kashan, Iran). XRD data were collected using Cu-Kα radiation (λ = 0.154 nm) at room temperature via an X-ray diffractometer (Philips X'Pert Pro MPD). Various two values ranging from 10° to 80° were examined in the XRD analysis. These techniques collectively provided insights into the nanocarriers' physical and chemical characteristics.

Scanning electron micrographs were acquired using FE-SEM TESCAN VEGA 3 and MIRA 3 instruments. The outcomes of the FE-SEM analysis provided insights into the morphology of the NPs, offering details on their surface characteristics and geometric structure. A small volume of the nanoparticle suspension was placed on carbon tape and air-dried at room temperature to prepare samples for imaging.

To quantify the amount of unencapsulated SFB (free drug) present in the samples, a UV-spectrophotometric technique at a wavelength of 265 nm was employed [4]. By utilizing a previously established calibration curve in PBS (pH 7.4) with a regression coefficient of 0.9793, the DL% and EE% of the samples were determined. The calculations for these two parameters were carried out using the equations established beforehand (Eqs. (1), (2))). These analyses allowed for the assessment of drug encapsulation and loading within the nanocarriers.

(1) $$EE\%=\frac{weight\;of\;loaded\;drug}{weight\;of\;total\;drug\;used}\times 100$$

(2) $$DL\%=\frac{weight\;of\;loaded\;drug}{weight\;of\;nanoparticles}\times 100$$

### 2.4 *In vitro* release study

A dialysis bag with a capacity of 2 mL was utilized to contain the sample under investigation. The dialysis tubing is subjected to chemical and physical treatments to enhance its resistance, using regenerated cellulose with a molecular weight cut-off (MWCO) ranging from 12,000 to 14,000 Da. The dialysis bag was securely sealed at both ends. It was then immersed in 100 mL of pH 7.4 PBS solution and maintained at three distinct temperatures: 37, 40, and 45 °C. The dispersion was gently stirred at 100 rpm using a magnetic stirrer.

At predefined intervals of 2, 4, 6, 24, 48, 72, 96, and 120 h (all at pH 7.4), 2 mL portions of the PBS solution were removed and replaced with an equal volume of fresh PBS. The drug concentration released into the PBS solution was measured using a UV-spectrophotometric technique at a wavelength of 265 nm. The calibration curves with a coefficient of determination (R^2^ = 0.9793) (Fig. 1S) were used to quantify the drug concentrations.

Each test was conducted in triplicate to ensure accuracy and consistency in the results. This in vitro release study provided insights into the controlled release behavior of the nanocarriers under varying conditions.

## 3 Results and discussion

A popular study area is biomaterials for creating and designing efficient drug delivery systems. Liposomes with different compositions have been incorporated into magnetic NPs or polymers to investigate their potential efficacy in drug molecules' targeted and controlled release from liposomes [[22], [23], [24],^59^]. These composed systems were developed to complete the advantages of polymeric and liposomal systems. The polymeric coating prevents drug molecules from interacting with their surroundings. Smart polymers may regulate the loading and release of encapsulated drugs in response to changes in the surrounding medium, including temperature [60]. Liposomal hydrogels, practical and multifunctional carriers, may be loaded with numerous chemotherapy drugs to treat local cancers for parenteral formulations. In such research, biocompatible nanocarriers consisting of a polymeric blend of thermosensitive PNIPAAm (synthesized and characterized using Free and RAFT techniques) and CS-coated magnetotocosome NPs were evolved to magnetically target the delivery of SFB anticancer drug and control its release in response to temperature changes. These nanocarriers were called CS-Free PNIPAAm-magnetotocosome and CS-Raft PNIPAAm-magnetotocosome, correspondingly. Different analyses characterized the designed systems to study their properties. The optimal composition for merging SPIO NPs was discovered after analyzing the impact of CHL molar ratio and drug concentration on tocosome particle size and drug loading. Table 1 displays the outcomes that were attained.

### 3.1 Investigation of CHL molar ratio and drug concentration effects on particle size and drug loading in tocosomes

**Fig. 3:**
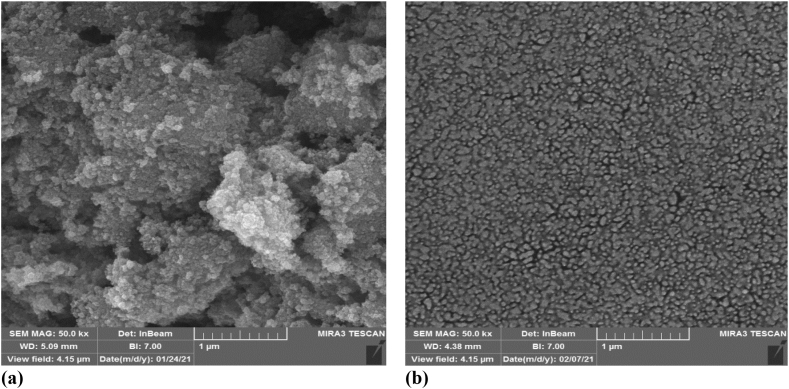
FE-SEM images of **a.** SPIO and **b.** PC-SPIO.

**Fig. 4:**
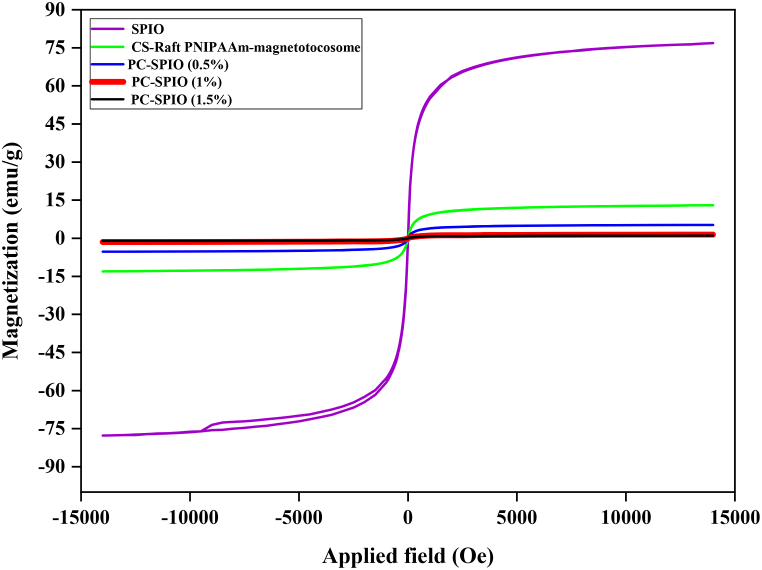
VSM results from synthesized samples.

**Fig. 5:**
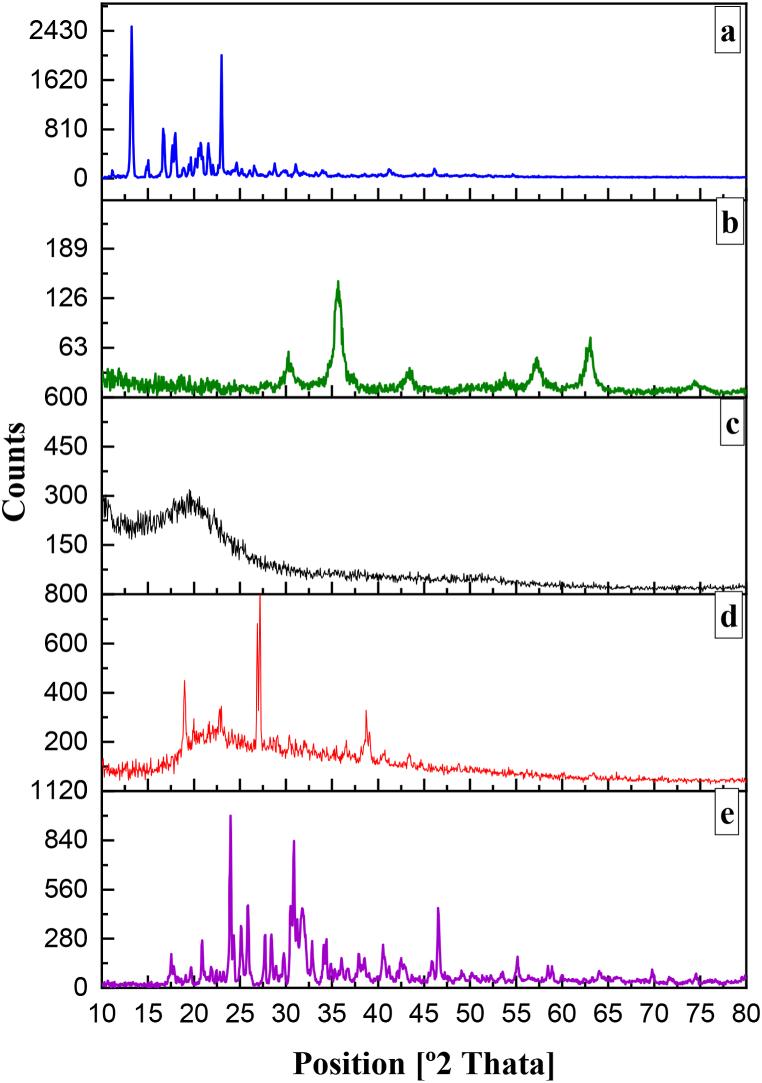
XRD analysis of **a.** pure SFB, **b.** SPIO, **c.** CS-Raft PNIPAAm, **d.** CS-Raft PNIPAAm-magnetotocosome and **e.** PBS.

The hydrodynamic size of particles plays a pivotal role in enhancing their accumulation within tumors. NPs with smaller diameters and higher specific surface areas can accelerate absorption rates and quantities, making them attractive for drug delivery applications [1,12,20]. Additionally, formulations containing smaller liposomes embedded in hydrogels have demonstrated superior sustained release profiles compared to larger multilamellar vesicles [23]. This study synthesized SFB-loaded tocosomes using Mozafari's technique, varying the CHL molar ratio and drug concentration. The resulting tocosomes exhibited an average diameter range of 64.9–147.8 nm (as shown in Table 1), indicating favorable colloidal stability of the NPs in aqueous environments**.** Furthermore, a satisfactory PDI was ascertained (approximately 0.2), thus implying a consistent distribution of NP sizes. NPs exhibit a pronounced inclination towards aggregation within colloidal solutions. The accumulation process introduces modifications to NP surface areas, thereby eliciting conformational alterations. This agglomeration phenomenon can be managed through strategies such as polymer coating or ultrasonication, as highlighted by prior scholarly work [48]. Supplementary materials encompassed graphical representations of particle size distribution and autocorrelation profiles (Fig. 2S–5S).

Prior investigations into liposome synthesis methodologies informed the selection of variables (molar ratio of CHL to drug and drug concentration) and their corresponding values [22,28,29,57]. Utilizing the data presented in Table 1, the influence of CHL molar ratio and drug concentration on particle size and DL was rigorously assessed via analysis of variance (ANOVA). Notably, the CHL molar ratio emerged as the parameter exerting the most significant impact on particle size (p = 0.0445). It was observed that the dimensions of tocosomes escalated at a specific threshold of drug concentration as the corresponding molar ratio expanded. Conversely, the impact of the drug concentration parameter (p = 0.1852) on tocosome particle size was deemed statistically insignificant.

Upon closer examination of Table 1, the EE% and DL% values for SFB ranged from 51.61 % to 77.88 % and from 2.59 % to 9.75 %, respectively (across various CHL molar ratios and drug concentrations). These findings suggest that tocosomes possess potential as a viable carrier for SFB.

The statistical analysis of the collected data revealed the relative significance of various factors that influence the responses of interest. The ANOVA results highlighted the importance of drug concentration (p = 0.0284) with DL. Notably, the molar ratio of CHL had no substantial effect on DL (p > 0.05). However, it was observed that increasing the drug concentration led to a noteworthy enhancement in DL, particularly at a specific CHL molar ratio.

As presented in Table 1, the stability of the synthesized tocosomes was assessed by evaluating their particle size (d_3_-d_0_) after a storage period of three months at a temperature of 4 °C. Based on the obtained values, it can be inferred that the tocosomes synthesized during this study exhibit favorable stability characteristics. Based on the considerations of tocosome size and DL, the optimal composition (sample 3) was chosen. Regarding the importance of the quantity of loaded drugs within a drug delivery system, the preferred sample exhibited a higher drug concentration. Additionally, as previously noted, an increase in the molar ratio of CHL led to an augmentation in the size of toco some particles. Consequently, a CHL variant with a lower molar ratio was utilized in this context.

### 3.2 Characterization of samples

**Fig. 6:**
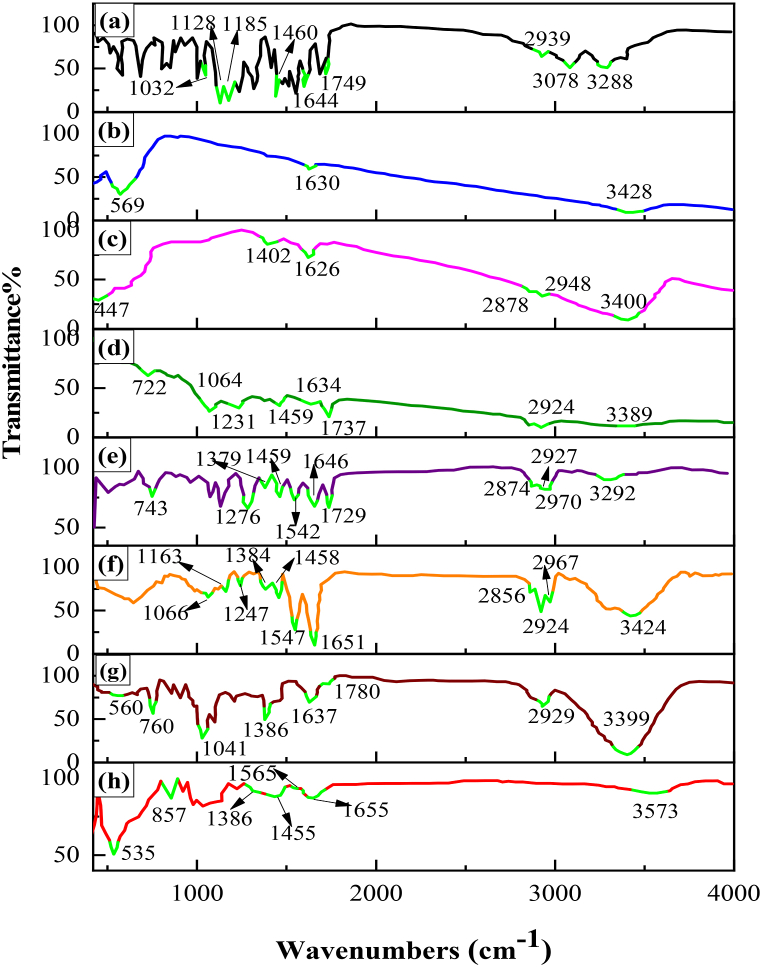
FTIR spectrum of **a.** original SFB, **b.** SPIO, **c.** PC-SPIO, **d.** pure PC, **e.** Raft-PNIPAAm, **f.** CS-Raft PNIPAAm, **g.** magnetotocosome and **h.** CS-Raft PNIPAAm-magnetotocosome.

**Fig. 7:**
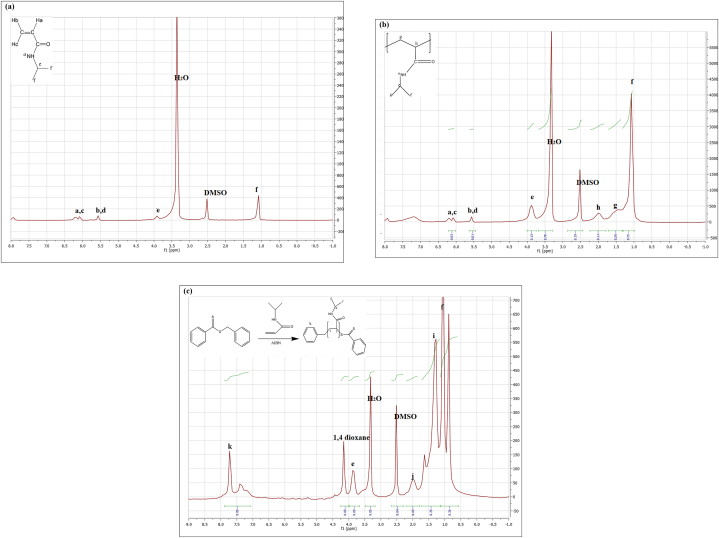
^1^H NMR spectra for **a.** NIPAM, **b.** Free-PNIPAAm and **c.** Raft-PNIPAAm.

The attainment of controlled SPIO precipitation is a prerequisite for achieving uniform particle size, a feat made feasible through the utilization of polymers or extended-chain surfactants such as Polycarbonate (PC) during the precipitation process [18]. The present study mitigated the crystalline nature of SPIO NPs by applying PC solutions at three distinct concentrations (with molar ratios of PC to iron ions set at 0.5 %, 1 %, and 1.5 %) during the preparation phase. PC integration throughout the procedure facilitated meticulous control over particle dimensions, culminating in the production of NPs characterized by reduced diameters, a narrower size distribution, and enhanced dispersion properties. Two plausible mechanisms come to the fore: (1) the chelation of these functional groups with metal ions, effectively impeding the formation of nuclei, and (2) the growth of nuclei potentially being restricted through the adsorption of these ions [48,19]. The discrepancy in particle size between standard SPIO and PC-treated SPIO becomes evident through the visual portrayal provided by FE-SEM images, as illustrated in Fig. 3a and b and DLS analysis in Figs. 6 and 7S. Henceforth, a conjecture emerges that the Polycarbonate (PC) concentration escalation during the precipitation process could impose constraints on particle growth, a consequence attributed to the augmented count of encapsulating molecules. This phenomenon could culminate in the formation of smaller particles distinguished by a more confined size distribution.

The decline in particle size, i.e. increase of surface, can be helpful in biological and medical applications but particles crystallinity is decremented by lowering the particles size below 10 nm (for many materials), hence, reducing the saturation magnetization. In the other hand, for drug delivery and hyperthermia applications, the saturation magnetization value of 8–19 emu.g^−1^ is required [61]. Therefore, particle size and saturation magnetization should be optimized for designing of magnetic drug delivery systems [16,18]. The magnetic characteristics of the prepared NPs were determined through VSM analysis conducted at room temperature. As the VSM results depicted in Fig. 4 indicate, the PC-SPIO NPs synthesized in this study exhibited saturation magnetization values of 5.24, 1.43, and 0.99 emus.g^−1^, corresponding to molar ratios of PC to iron ions of 0.5 %, 1 %, and 1.5 %, respectively. These values fell short of the requisite range for biomedical applications. Consequently, the decision was made to employ SPIO NPs in the formulation of magnetotocosomes.

The magnetization curves depicted in Fig. 4 demonstrated the absence of coercivity and remanence, thereby confirming the superparamagnetic attributes of the NPs. The saturation magnetization value for the SPIO NPs registered at 76.8 emus.g^−1^ and experienced a reduction to 12.96 emus.g^−1^ in the CS-Raft PNIPAAm-magnetotocosome formulation. This diminution could be attributed to the effective encapsulation of the NPs by the toco some layer and the composite composition comprising CS and PNIPAAm. Moreover, the SPIO NPs within the polymeric carrier could encounter challenges in aligning themselves along their preferred axis of magnetization, contributing to the reduction in saturation magnetization [61].

The noteworthy saturation magnetization value, coupled with the superparamagnetic nature of the nanocarriers conceived in this study, renders them highly promising candidates for applications in drug delivery and magnetic hyperthermia, a therapeutic approach where cancer cells can be targeted and eliminated through exposure to temperatures around 42–45 °C [48]. Furthermore, these nanocarriers can potentially address a significant challenge associated with SFB: the difficulty in confining drug cytotoxicity exclusively to the intended target region. Incorporating SPIO NPs within the tocosome structure imparts magnetic properties to the nanocarriers, allowing for controlled movement and targeting upon exposure to static magnetic fields.

To assess the crystallinity of the different entities, namely SFB powder, SPIO, the CS-Raft PNIPAAm polymer blend, CS-Raft PNIPAAm-magnetotocosome, and PBS, XRD analysis was conducted. The resultant diffraction patterns for these substances are presented in Fig. 5. The diffraction pattern for pure SFB (Fig. 5a) exhibited distinct characteristic peaks at 13.2°, 15°, 16.5°, 17.8°, and 23.1°, indicative of a high degree of crystallinity.

SPIO NPs' XRD pattern is shown in Fig. 5b.

The XRD analysis of these NPs exhibited distinct peaks at angles of 0.1°, 35.5°, 43.1°, 53.5°, 57.1°, and 62.8°, corresponding to the reflection plane index values (2 2 0), (3 1 1), (4 0 0), (4 2 2), (5 1 1), and (4 4 0), respectively. These reflections signify the presence of well-defined crystalline planes within the NPs, specifically reflecting their crystallographic structure [20,48]. These observations indicate that the sonicated SPIO NPs possess satisfactory quality and purity. The narrow and well-defined nature of the SPIO peaks further suggests that these NPs exhibit a relatively elevated degree of crystallinity.

The absence of maghemite (γ-Fe_2_O_3_) traces was inferred from the absence of (210) and (300) peaks in the XRD spectra. The coating process was demonstrated by a reduction in the intensity of the SPIO peaks, as evidenced in the XRD pattern of the CS-Raft PNIPAAm-magnetotocosome formulation (Fig. 5d). The distinct, sharp peaks attributed to PBS in the XRD pattern of the CS-Raft PNIPAAm-magnetotocosome formulation rendered the SPIO-related peaks relatively inconspicuous. This observation suggests that the crystal phase of the magnetic NPs remained largely unaltered by the coating process. This alignment with prior research findings supports the consistency of this outcome [13,20]. The XRD pattern corresponding to PBS is depicted in Fig. 5e. The Scherrer formula determines crystallite size from X-ray line broadening (Eq. (3)):

(3) D=0.9λ/βcosθ

D determines the average crystalline size (D), with λ = 0.154 nm representing the X-ray wavelength, β indicating the angular line width at half maximum intensity, and θ representing Bragg's angle [18,19]. The estimated crystallite size for the SPIO is 12 nm, implying that these particles are suitable for targeted drug delivery applications. Fig. 5c showcases the XRD patterns for the composite of CS and Raft-PNIPAAm. A broad diffraction peak at 20.8° verifies the existence of CS with low crystallinity, attributed to its layered structure [33].

The elevated melting point of the drug accounts for its limited solubility in aqueous environments. Hence, strategies aimed at disrupting crystallinity or reducing crystal lattice energy (such as dispersing the drug within a polymeric carrier) can lead to partial or complete amorphization of the drug, consequently enhancing its aqueous solubility [8]. Drawing a comparison from the XRD analysis presented in Fig. 5, it becomes apparent that the CS-Raft PNIPAAm-magnetotocosome formulation may impede drug crystallization by facilitating the creation of amorphous solid dispersions.

In the FTIR spectra of the drug (Fig. 6a), characteristic bands related to various bonds, including C–C (Stretching), C–O (Stretching), C=O (Stretching), C–NH (Aromatic Stretching), CH_3_ (Bending), and OH (Bending) bonds were observed at wavenumbers of 1032 and 1460 cm^−1^, 1128 cm^−1^, 1749 cm^−1^, 1644 cm^−1^, and 2939 and 3288 cm^−1^, respectively [6]. Additionally, bands associated with stretching vibrations of C–F bonds were discernible in the FT-IR spectra at a wavenumber of 1185 cm^−1^ [62].

**Fig. 8:**
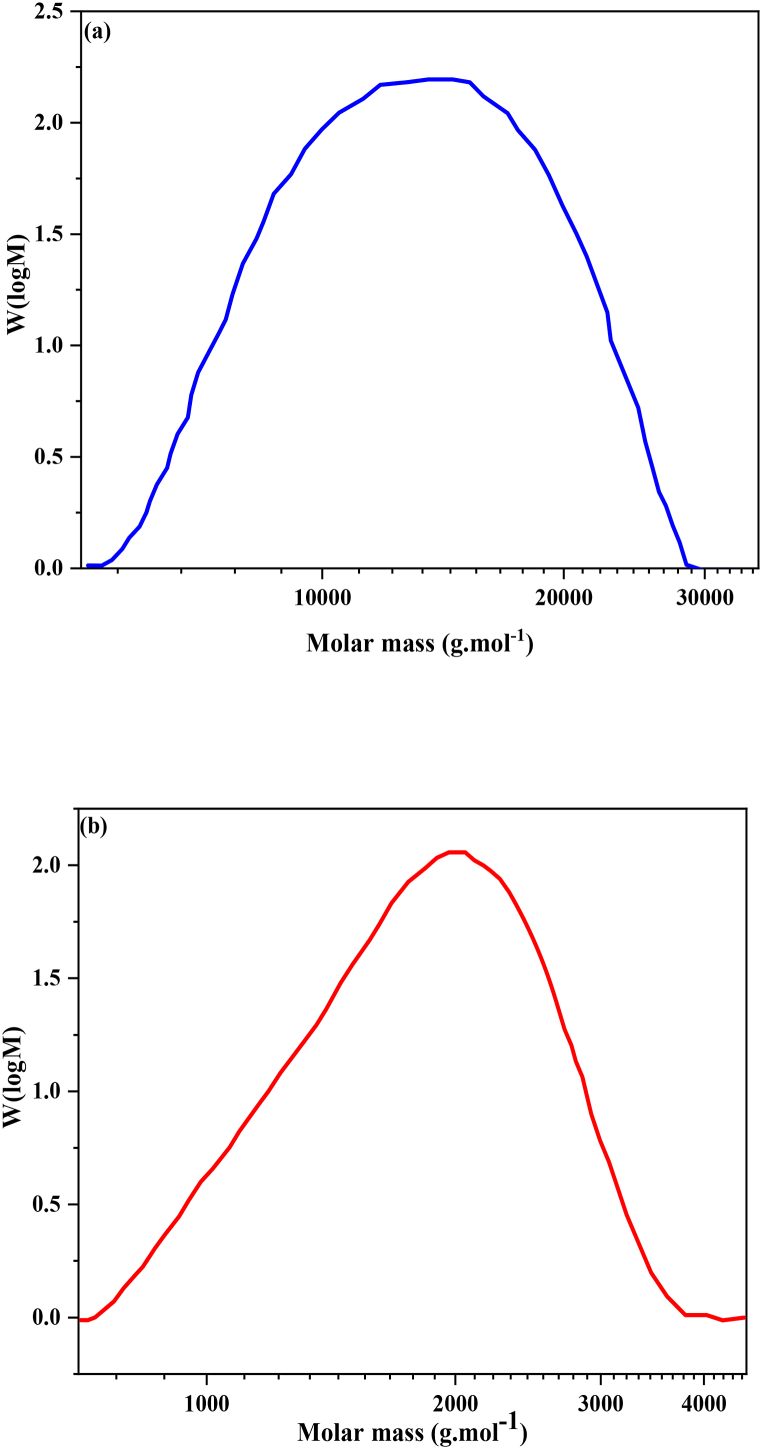
GPC results for **a.** Free-PNIPAAm and **b.** Raft-PNIPAAm.

**Fig. 9:**
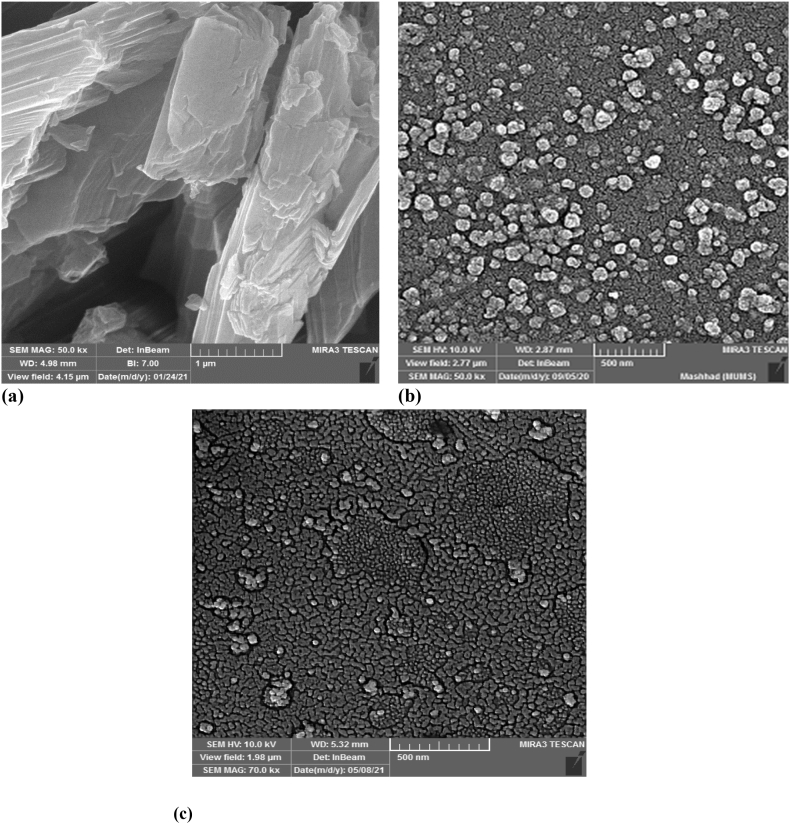
FE-SEM images of **a.** original SFB **b.** tocosome suspension (sample 3), **c.** CS-Raft PNIPAAm-magnetotocosome.

In the FT-IR spectrum of SPIO (Fig. 6b), three notable absorption bands were observed at 3428 cm^−1^, 1630 cm^−1^, and 569 cm^−1^. The bands at 3428 cm^−1^ and in the range of 1620–1630 cm^−1^, attributed to O–H stretching and H–O–H bending modes, respectively, indicate the presence of adsorbed water. The interactions involving oxygen atoms in octahedral and tetrahedral positions contribute to the appearance of these stretching vibrations. In the PC-SPIO spectrum (Fig. 6c), distinct bands were identified at wavenumbers 2878 cm^−1^ and 2948 cm^−1^, corresponding to symmetric and asymmetric methylene (–CH_2_) and methyl (–CH_3_) vibrations, respectively. The band observed at 1402 cm^−1^ is attributable to the bending vibration of the –CH_2_ group. Notably, in addition to the SPIO-specific bands, the PC-SPIO spectrum includes bands characteristic of pure PC (Fig. 6d), indicating the presence of adsorbed PC on the surface of the SPIO NPs [19]. The NIPAM spectrum exhibited a peak at 1619 cm^−1^, attributed to the absorption of the –C=C-vinyl group (Fig. 8S). This signal, notably absent in the Raft-PNIPAAm spectrum (Fig. 6e), signifies successful polymerization and purification processes. The stretching vibration peaks corresponding to amide I and II (observed at 1646 cm^−1^ and 1542 cm^−1^) remained unchanged following polymerization. The C–H stretching bands manifested at three distinct positions: 2970 cm^−1^ (isopropyl –CH_3_ asymmetric stretch), 2927 cm^−1^ (acrylamide backbone –CH_2_ asymmetric stretch), and 2874 cm^−1^ (isopropyl –CH_3_ symmetric stretch). In contrast, the peaks situated at 1459 cm^−1^ (asymmetric deformation of isopropyl –CH_3_) and 1379 cm^−1^ (symmetric deformation of isopropyl –CH_3_) are associated with C–H deformation [32,63]. A signal at 1729 cm^−1^, corresponding to 1,4-dioxane, indicates that the specimen was not entirely moisture-free. After a further drying process, the peak at 1729 cm^−1^ was eliminated from the FTIR spectra of the CS-Raft-PNIPAAm mixture (Fig. 6f). The supplementary material encompasses the FT-IR spectrum of PNIPAAm synthesized through free radical polymerization (Free-PNIPAAm) (Fig. 9S). FTIR spectra were employed to investigate the interactions of the magnetotocosome with a blend of CS and PNIPAAm. The distinctive peaks associated with the magnetotocosome, CS-Raft-PNIPAAm-magnetotocosome, and the composite of CS and Raft-PNIPAAm were examined. Notably, a weak-intensity peak at 1780 cm^−1^ corresponds to the stretching vibration of the C=O bond in the ester linkage, where the fatty acid chain interfaces with the head group in all liposomal dispersions. The symmetric and antisymmetric stretching vibrations of CH_2_ groups (2800-3000 cm^−1^) and absorption at 3399 cm^−1^ arise from the stretching vibrations of OH and NH functional groups. Additionally, characteristic peaks associated with SPIO NPs (1637 cm^−1^ and 560 cm^−1^) were discernible in this FTIR spectrum (Fig. 6g) [22]. The frequencies attributed to various groups, such as C–H bending from NIPAM methyl groups and amide III vibrations from CS, combined to form a consolidated band at 1384 cm^−1^ in the spectra of the CS and Raft-PNIPAAm blend (Fig. 6f). The peak at 3424 cm^−1^ was attributed to the stretching vibrations of the -NH2 and –OH groups. Additionally, the vibrational mode at around 1066 cm^−1^ corresponds to the symmetric stretching of the (C–O–C) bond of the glucosamine unit linked to the CS monomer. The peak at 1163 cm^−1^ corresponds to the asymmetric stretching of the glycosidic bond between the two monosaccharides in CS. Peaks at 1651 cm^−1^ and 1547 cm^−1^ correspond to the amides I and II, respectively. Peaks at 1458 cm^−1^ (-CH_3_) and 1651 cm^−1^ (O=C) confirmed the presence of PNIPAAm moieties. Notably, the interaction between the blend and magnetotocosome was evidenced by a shift in the C=O band from approximately 1780 cm^−1^ in the magnetotocosome to 1655 cm^−1^. Another interaction within the blend of CS and Raft-PNIPAAm was indicated by a shift from 3424 cm^−1^ (associated with OH and NH regions in the blend) to 3573 cm^−1^ (an apparent peak) in the CS-Raft PNIPAAm-magnetotocosome (Fig. 6h). The characteristic peaks also corroborated the presence of the PNIPAAm polymer at 1455 cm^−1^ (-CH_3_) and 1655 cm^−1^ (O=C). Short peaks indicative of amide II and III could be discerned around 1565 cm^−1^ and 1386 cm^−1^, respectively. The ^1^H NMR spectra of NIPAM, Free-PNIPAAm, and Raft-PNIPAAm have been presented in Fig. 7. Peaks associated with hydrogens adjacent to double-bonded carbons were observed in the 5.5–6.5 ppm range. These same peaks were evident in the NIPAM spectrum, indicating the presence of three such entities in NIPAM (Fig. 7a). These observations confirm the synthesis of a PNIPAAm homopolymer (Fig. 7b and c). Moreover, the FTIR data suggests that the peaks at 4 ppm and 5 ppm in the Raft-PNIPAAm spectrum could be attributed to the presence of the 1,4-dioxane solvent. The rest of the CTA was removed in the precipitation step. Any peaks related to CTA were not observed in FTIR and NMR results.

GPC analysis was employed to assess the molecular mass distribution of the synthesized PNIPAAm. The outcomes of this analysis are presented in Fig. 8a and b. The MWs and PDIs of Free-PNIPAAm and Raft-PNIPAAm were determined to be13825 and 1.13, and 1957 and 1.12, respectively. Polydispersity values in the range of 1.2–1.4 are generally deemed acceptable indicators of control over the MW distribution of PNIPAAm polymers. Notably, RAFT polymerization yielded PDI values of approximately 1.13 or even lower, indicative of notably narrow MW distributions.

It's important to highlight that Raft-PNIPAAm exhibits a notably low MW, and this attribute significantly impacts the LCST of the polymer. The LCST can be influenced by the length of polymer chains and their concentration. Increasing the MW and polymer concentration generally results in a reduction of the LCST [64]. At a given temperature, higher concentrations of PNIPAAm lead to stronger hydrophobic interactions among the chains, resulting in larger aggregates and increased turbidity [56]. Enhanced hydrophobic interactions simplify the formation of aggregates at lower temperatures.

**Fig. 10:**
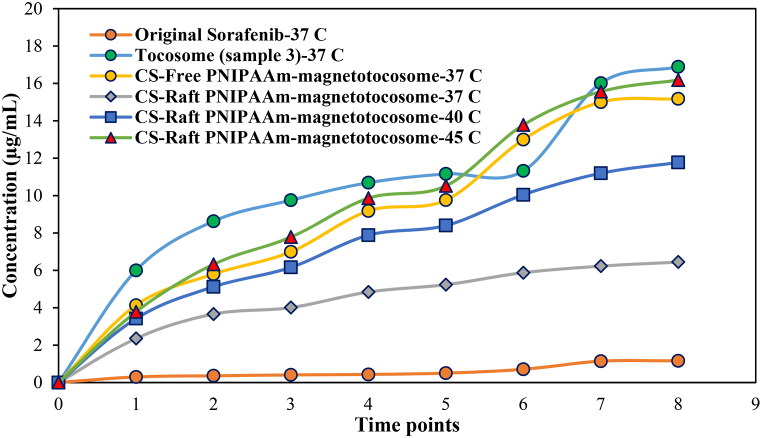
The release profiles of SFB from designed drug delivery systems at different times (1: 2, 2: 4, 3: 6, 4: 24, 5: 48, 6: 72, 7:96, 8: 120 h).

Researchers have observed the coil-to-globule transition of PNIPAAm using DSC analysis [24,34,63,65,66]. An endothermic peak is observed in the DSC thermograms, indicating precipitation in these systems (Fig. 10S). The DSC curves for the blend of CS and Raft-PNIPAAm, as well as CS Free-PNIPAAm, revealed the efficient transformation of hydrophilic polymer chains into hydrophobic conformations at 45 °C and 33 °C, respectively. The observation that polymers with higher MWs exhibit lower LCSTs underscores the significance of chain length in PNIPAAm's phase transition. Longer polymer chains and higher concentrations lead to lower cloud points [64]. In this case, the LCST values of the CS/Raft-PNIPAAm combination were higher than the typical body temperature. Localized hypothermia, a documented cancer therapy approach, involves concentrating the medication around the tumor. Conventional techniques often require daily doses through sustained-release tablets. Thus, the focus has shifted towards responsive drug delivery systems that can control drug release rates in response to external stimuli [33]. Mild hyperthermia, up to 44 °C, can be tolerated by body tissues for extended periods without causing irreversible damage. Hyperthermia techniques such as microwaves, ultrasonic waves, radiofrequency, or magnetic fluid hyperthermia [65] could be harnessed for this purpose.

To validate the presence of magnetotocosomes within the polymeric blends, the morphology, and structure of tocosomes and polymer-coated magnetotocosomes were examined using FE-SEM (Fig. 9). The drug crystals exhibited a range of shapes and sizes, as seen in Fig. 9a. The SEM image in Fig. 9b depicts a suspension of tocosomes (sample 3) displaying distinct spherical structures, as anticipated for lipid vesicles with a 3D arrangement. Similarly, Fig. 9c reveals the presence of tocosomes within the CS and Raft-PNIPAAm blend. Importantly, the polymer-coated magnetotocosomes did not tend to agglomerate. Furthermore, the use of the polymer as a coating agent did not compromise membrane stability, as evidenced by the maintained spherical form and membrane integrity of the tocosomes despite the polymer coating.

Table 1 illustrates the zeta potentials of both tocosomes and polymer-coated magnetotocosomes, with the values representing the averages from three measurements. The tocosomes exhibited relatively low zeta potentials. Table 1 provides further details on the zeta potential measurements and particle size analysis for the polymer-coated magnetotocosome. The table reveals a gradual increase in particle diameters due to polymer coating. Supplementary information presents DLS curves (Fig. 11S–14S).

Upon introducing polymeric blends, the zeta potential of tocosomes (sample 3, -19.1 mV) shifted to approximately −13.3 mV and −15 mV for CS-Free PNIPAAm-magnetotocosome and CS-Raft PNIPAAm-magnetotocosome, respectively. These zeta potential values closely resemble what is expected from a pure mixture of CS and PNIPAAm. This finding indicates that the polymeric blend effectively covers the surfaces of magnetotocosomes. The complete deprotonation of CS amino groups results in the CS exhibiting a negative charge in the PBS solution at pH 7.4. Fig. 1 presents the chemical structure of SFB, which contains amine and pyridine groups with pKa values of 12.89 and 2.60, respectively. In a neutral medium, these functional groups can form hydrogen bonds with CS [20]. Analyzing the calculated values of EE%—97.2 % for CS-Free PNIPAAm-magnetotocosome and 98.8 % for CS-Raft PNIPAAm-magnetotocosome—along with DLvalues of 1.21 % and 1.24 % for these systems, respectively, as well as the stability of polymer-coated magnetotocosomes, the developed drug delivery system displays promising potential for encapsulating bioactive agents. The procedure for creating magnetotocosomes was safe and scalable, conducted at 65 °C without toxic solvents or detergents. As a result, the polymer-coated magnetotocosome could serve as a versatile and effective multi-objective drug delivery system.

### 3.3 The release of SFB from both tocosomes and polymer-coated magnetotocosomes at various temperatures

Due to their capacity for self-assembly and subsequent transformation into hydrogels upon injection, polymers represent a promising alternative for coating liposomal pharmaceutical compounds, facilitating sustained administration across various medical applications. This investigation proposes using polymer-coated magnetotocosomes as nanocarriers to modulate the distribution of SFB within tumor blood vessels.

Fig. 10 delineates the release of SFB from the synthesized samples in a PBS solution (pH = 7.4) at various temperatures, contingent upon the polymeric blends' LCST. The figure illustrates that approximately 8.5 % of the initial SFB content was released into the medium within the first 2 h at 37 °C, followed by a sustained release over 48 h. Following this period, the release rate slightly increased, culminating in a drug concentration of 1.16 μg/mL (33.2 %) at 120 h. This relatively slow dissolution rate can

be attributed to the drug's low aqueous solubility.

Both Tocosome (sample 3) and CS-Free PNIPAAm-magnetotocosome exhibited distinct release profiles for the initial SFB content. They released the drug with concentrations of approximately 5.99 μg/mL and 4.13 μg/mL (28.2 % and 20.7 % of their loaded drug, respectively) within the initial 2 h, followed by an escalating release rate of up to 96 h. The data indicates that within 120 h, the drug was released into the PBS solution at 16.88 μg/mL and 15.17 μg/mL (equating to 79.5 % and 75 % of the total drug, respectively) for both specimens.

The release of SFB involves drug diffusion and the degradation of the polymer coating. Drug molecules near the surface also tend to exhibit a faster initial release rate.

The findings suggest that tocosomes and CS-Free PNIPAAm-magnetotocosome could serve for sustained drug administration but may not be suitable for hyperthermia applications. On the other hand, CS-Raft PNIPAAm-magnetotocosome demonstrated a tightly retained SFB content when exposed to temperatures of 37 °C (below the LCST of the polymeric blend), releasing only 42.9 % of its content (with a concentration of 6.44 μg/mL) over 120 h. The SFB release profile exhibited significant increments at temperatures higher than 37 °C.

CS-Raft PNIPAAm-magnetotocosome showcased interesting release behavior. At 40 °C (close to the LCST), it released the drug with concentrations of 3.42 μg/mL (17.1 % of SFB) within 2 h. At 45 °C (the LCST), it released 3.78 μg/mL (18.9 % of SFB) within the same time frame (Fig. 10). The final drug concentrations from CS-Raft PNIPAAm-magnetotocosome at 40 °C and 45 °C were 11.77 μg/mL (58.8 % of loaded drug) and 16.18 μg/mL (80.9 % of loaded drug into the PBS), respectively, after 120 h.

The aggregation initiation temperatures and SFB release of the polymer-coated magnetotocosomes in PBS were lower than the LCST of the polymeric blend in water. This indicates that the polymeric blend's temperature-responsive behavior initiated the destabilization of polymer-coated magnetotocosomes and subsequent drug release. The hydrated polymeric blend chains provided stability to the tocosomes' coating on the magnetotocosome surface below the LCST. However, as the polymeric blend chains were dehydrated, they coated parts of the tocosomal surface above the LCST, reducing magnetotocosome stability.

## 4 Conclusion

SFB, the sole approved targeted treatment for hepatocellular carcinoma, faces several limitations, such as a narrow therapeutic window, poor water solubility, and hepatic first-pass effects that diminish its bioavailability. Addressing these limitations, a hybrid drug delivery system targeting tumor tissues emerges as a potential solution. This system can enhance SFB's solubility, pharmacokinetic behavior, and overall bioavailability while minimizing adverse effects.

The study adopts a theranostic approach, employing SPIO NPs loaded within liposomes (magnetoliposomes) as carriers for the magnetic targeting of drugs. In this research, a polymeric combination comprising CS and thermosensitive PNIPAAm is employed to coat magnetotocosomes—liposomes with an SPIO core. This strategic development transforms the magnetotocosome into an intelligent and biocompatible nanocarrier, boasting improvements such as elevated drug EE% of up to 98.8 %, extended stability, favorable particle size, and the potential for scalable industrial production. This innovative approach facilitates controlled drug delivery of SFB to cancerous tissues.

Utilizing a solvent-free technique proposed by Mozafari, tocosomes—vesicles with a lipid bilayer—are synthesized in a reliable and scalable manner. The impact of CHL molar ratio and drug concentration on tocosome characteristics—such as particle size, zeta potential, EE%, and DL—is meticulously considered. This assessment culminates in identifying an optimal composition for generating the magnetotocosome, further integrated with the polymeric blend.

In conclusion, this study presents a comprehensive strategy to address the limitations of SFB in hepatocellular carcinoma treatment through a hybrid drug delivery system. By leveraging the advantageous properties of magnetoliposomes and employing a novel polymeric blend, the research offers the potential for enhanced therapeutic outcomes in cancer therapy.

The CHL molar ratio played a pivotal role in determining particle size, with a noticeable impact on this characteristic. Specifically, an increase in the CHL molar ratio at a particular drug concentration led to an enlargement in tocosome size. On the other hand, drug concentration emerged as the primary factor influencing DL. Enhancing drug concentration, particularly at a specific CHL molar ratio, led to a significant rise in DL. Furthermore, the role of PC in influencing the synthesis and characteristics of SPIO NPs was investigated. During the precipitation process, PC was observed to exert a regulatory effect on particle development, producing smaller particles with a more confined size distribution. However, as the particle size decreased, the crystallinity of the NPs also decreased, subsequently affecting the concentration of magnetization.

The synthesis of a PNIPAAm homopolymer was accomplished using both the free radical and RAFT polymerization methods. The study delved into the impact of variables such as chain length, PDI, and polymer MW on the LCST of PNIPAAm. Characterization of the samples was conducted through techniques such as DLS, ^1^H NMR, FTIR, DSC, FE-SEM, GPC, VSM, XRD, and UV–Vis spectroscopy. The developed nanocarrier, CS-Raft PNIPAAm-magnetotocosome, exhibited an apparent LCST of approximately 45 °C. This nanocarrier can potentially induce magnetic hyperthermia and release drug payloads in a spatiotemporal manner. Remarkably, this nanocarrier released 80.9 % of the loaded drug into PBS over 120 h at a temperature of 45 °C.

## Data availability statement

Data will be made available on request.

## CRediT authorship contribution statement

**Fariba Razmimanesh:** Investigation, Methodology, Writing – original draft. **Gholamhossein Sodeifian:** Funding acquisition, Investigation, Methodology, Supervision, Validation, Writing – original draft, Writing – review & editing.

## Declaration of competing interest

The authors declare that they have no known competing financial interests or personal relationships that could have appeared to influence the work reported in this paper.

## Abbreviations

α-tocopheryl phosphate: TP
Analysis of variance: ANOVA
**Azobisisobutyronitrile**: AIBN
Ammonium persulfate: APS
Chitosan: CS
Cholesterol: CHL
Differential scanning calorimetry: DSC
Dynamic light scattering: DLS
Drug loading: DL
Field emission scanning electron microscopy: FE-SEM
Encapsulation efficacy: EE
Fourier transform infrared spectroscopy: FTIR
Gel permeation chromatography: GPC
^1^H nuclear magnetic resonance: ^1^H NMR
Lower critical solution temperature: LCST
Molecular weight: MW
*N-*isopropylacrylamide: NIPAM
Phosphatidylcholine: PC
Polydispersity index: PDI
Poly(N-isopropylacrylamide): PNIPAAm
Reversible addition-fragmentation chain transfer: RAFT
Sodium bisulfite: SBS
Sorafenib tosylate: SFB
Superparamagnetic iron oxide: SPIO
*Ultraviolet–visible*: UV–Vis
Vibrating sample magnetometer: VSM
X-ray diffraction: XRD
