# Supplementary material for: Evaluation of a temperature-responsive magnetotocosome as a magnetic targeting drug delivery system for sorafenib tosylate anticancer drug
Source: Heliyon. 2023 Nov 4;9(11):e21794. doi: 10.1016/j.heliyon.2023.e21794 (PMC10658271; doi:10.1016/j.heliyon.2023.e21794)
Supplement: Multimedia component 1 [file mmc1.docx]

**Investigation of temperature-responsive magnetotocosome as the magnetic targeting drug delivery system for Sorafenib tosylate anticancer drug: Effects of MW and chain length of PNIPAAm on LCST and dissolution rate**

**Fariba Razmimanesh ^a,b,c^, Gholamhossein Sodeifian ^a,b,c,*^**

*^a^Department of Chemical Engineering, Faculty of Engineering, University of Kashan, Postal Code: 87317-53153, Kashan, Iran.*

*^b^Laboratory of Supercriritcal Fluids and Nanotechnology, University of Kashan, Postal Code: 87317-53153, Kashan, Iran.*

*^c^Biotechnology Centre, Faculty of Engineering, University of Kashan, Postal Code: 87317-53153, Kashan, Iran.*

*Corresponding author. Tel.: +983155912406; fax: +98315591*2*424.

E-mail address: **sodeifian@kashanu.ac.ir (**G. Sodeifian**)**

**Fig. 1S. The calibration curve for SFB in PBS**

| **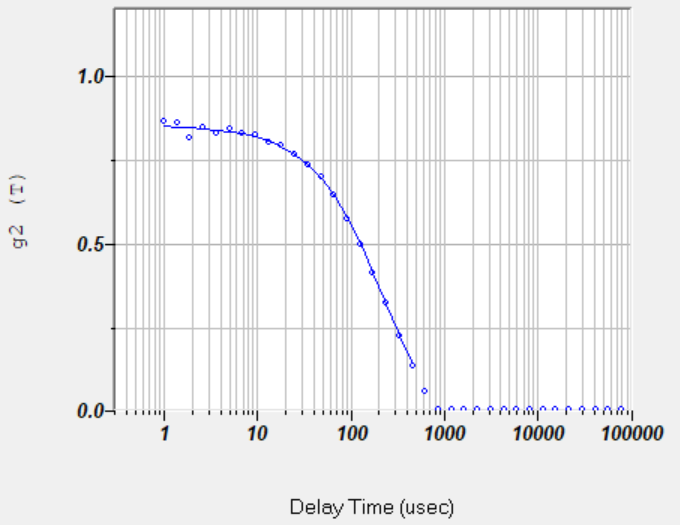** |  |
| --- | --- |

**Fig. 2S.** DLS and Autocorrelation curves for sample 1 (tocosomal dispersion).

| **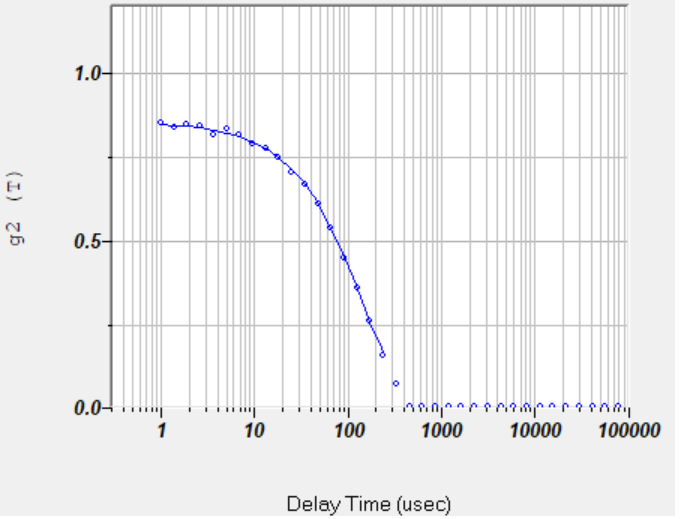** | **** |
| --- | --- |

**Fig. 3S.** DLS and autocorrelation curves for sample 2 (tocosomal dispersion).

| **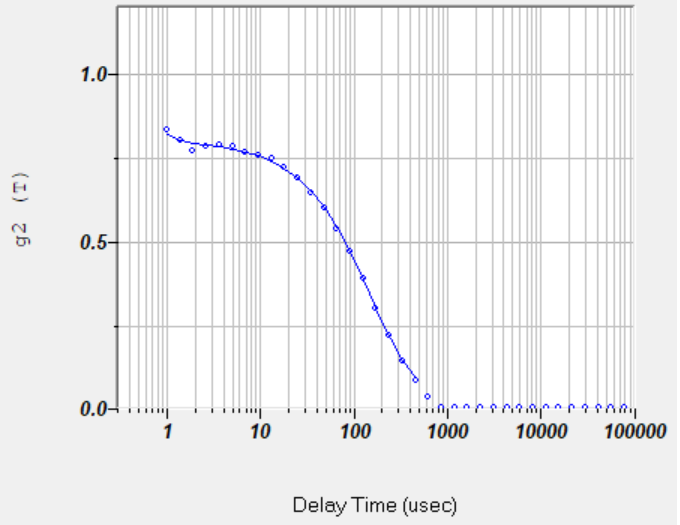** | **** |
| --- | --- |

**Fig. 4S.** DLS and autocorrelation curves for sample 3 (tocosomal dispersion).

| **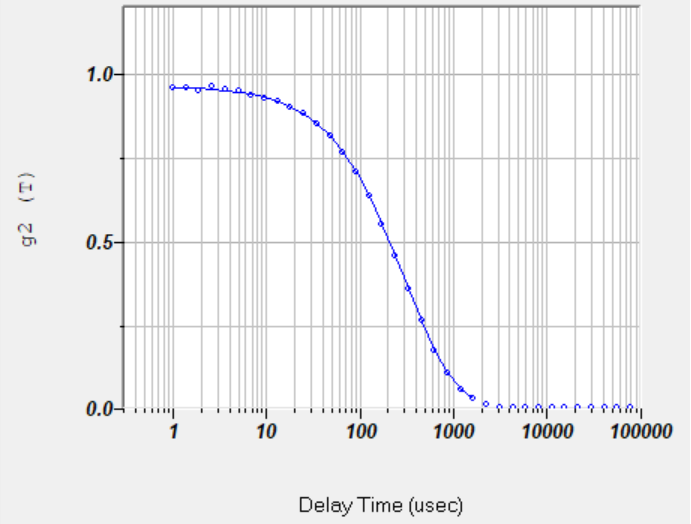** | **** |
| --- | --- |

**Fig. 5S.** DLS and autocorrelation curves for sample 4 (tocosomal dispersion).

| **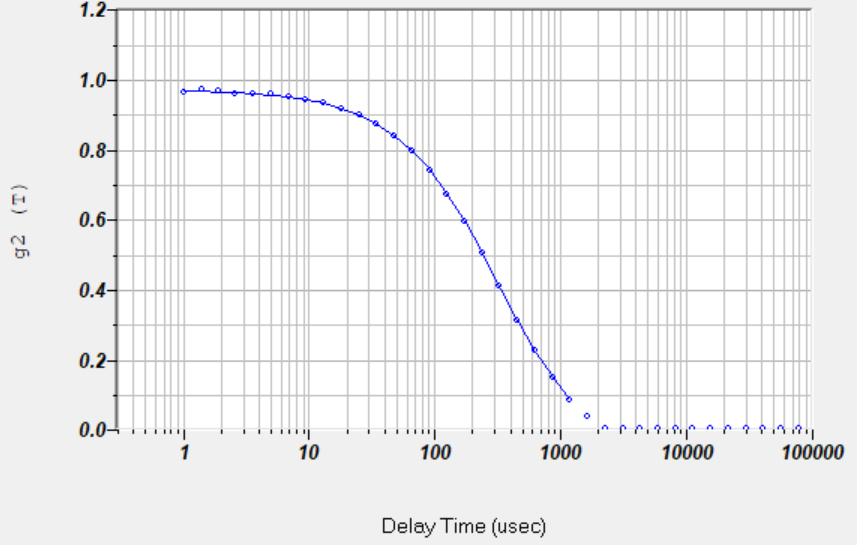** | **** |
| --- | --- |

**Fig. 6S.** DLS and autocorrelation curves for SPIO.

| **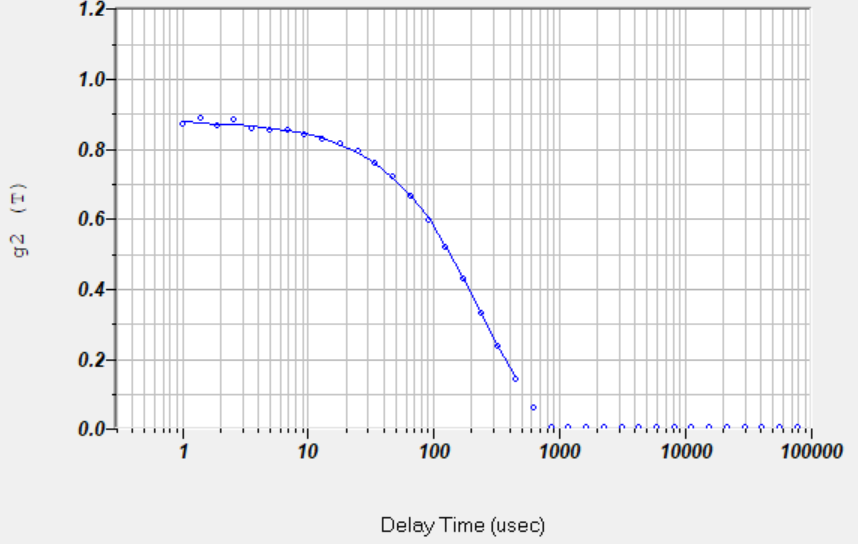** | **** |
| --- | --- |

**Fig. 7S.** DLS and autocorrelation curves for PC-SPIO.

**Fig. 8S.** FTIR spectrum for NIPAM.

**Fig. 9S.** FTIR spectrum for Free-PNIPAAm.

|  |
| --- |
|  |

**Fig. 10S.** DSC results for **a.** blend of CS and Free-PNIPAAm and **b.** blend of CS and Raft-PNIPAAm.

| **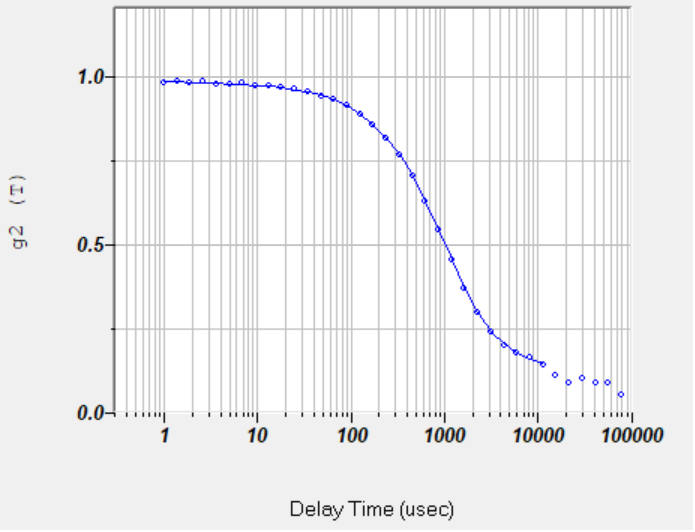** | **** |
| --- | --- |

**Fig. 11S.** DLS curve for CS-Free PNIPAAm-magnetotocosome.

| 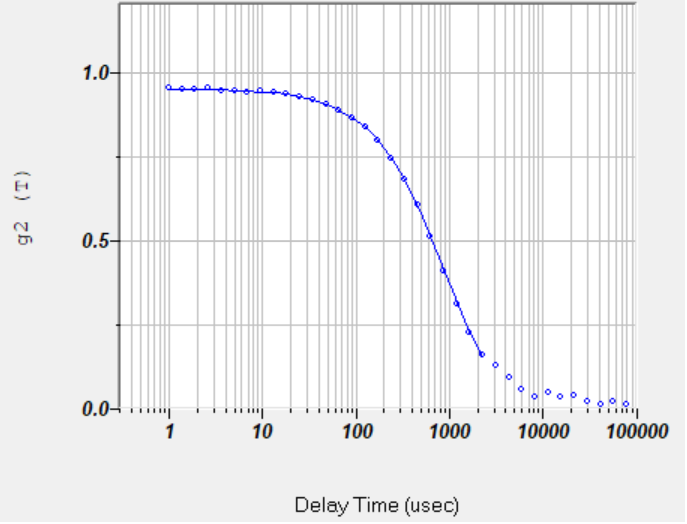 |  |
| --- | --- |

**Fig. 12S.** DLS curve for CS-Raft PNIPAAm-magnetotocosome.

| 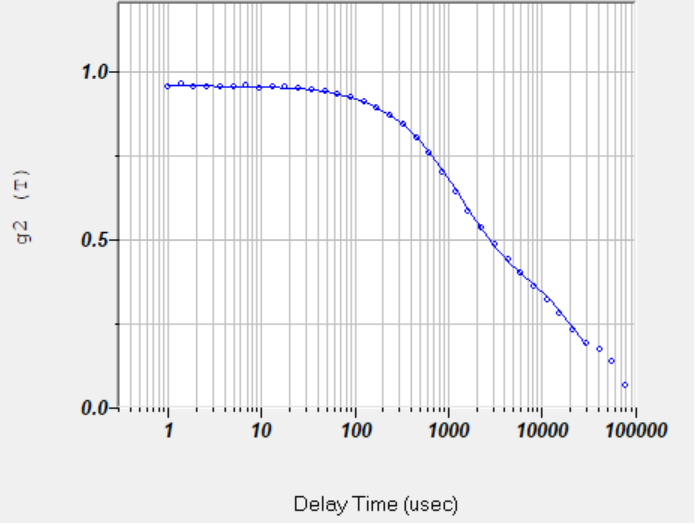 |  |
| --- | --- |

**Fig. 13S.** DLS curve for CS-Free PNIPAAm-magnetotocosome after 3 months.

| 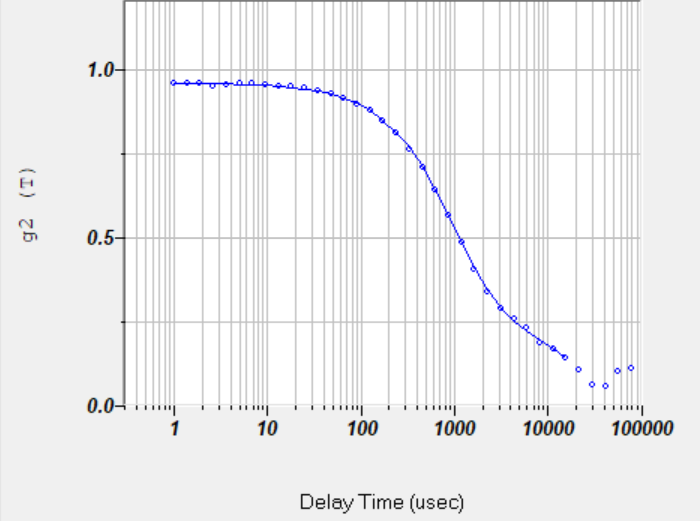 |  |
| --- | --- |

**Fig. 14S.** DLS curve for CS-Raft PNIPAAm-magnetotocosome after 3 months.
